# Hemangioma related to Maffucci syndrome in a man: a case report

**DOI:** 10.1186/1752-1947-5-224

**Published:** 2011-06-21

**Authors:** Takeshi Kondo

**Affiliations:** 1 Division of Legal Medicine, Department of Community Medicine and Social Healthcare Science, Kobe University Graduate School of Medicine, 7-5-1 Kusunoki-cho, Chuo-ku, Kobe 650-0017, Japan

## Abstract

**Introduction:**

Maffucci syndrome is a rare clinical entity (approximately 200 cases have been reported in the medical literature) with a combined occurrence of multiple enchondromas and vascular tumors.

**Case presentation:**

The case of a 43-year-old Japanese man with multiple chondromas and hemangiomas (Maffucci syndrome) is reported. One of the hemangiomas was removed and examined pathologically. The morphological picture was an admixture of cavernous hemangioma and spindle cell hemangioma without cytological atypia or mitosis. Sheets of vacuolated endothelial cells were also observed.

**Conclusion:**

A rare case of hemangioma associated with Maffucci syndrome, focusing on the pathologic nature of the submitted tissue, is reported.

## Introduction

Maffucci syndrome is a rare clinical entity (approximately 200 cases have been reported in the medical literature) [1]. It consists of combined occurrence of multiple enchondromas and vascular tumors. This syndrome is not inherited and shows female predilection.

## Case presentation

**Figure 1:**
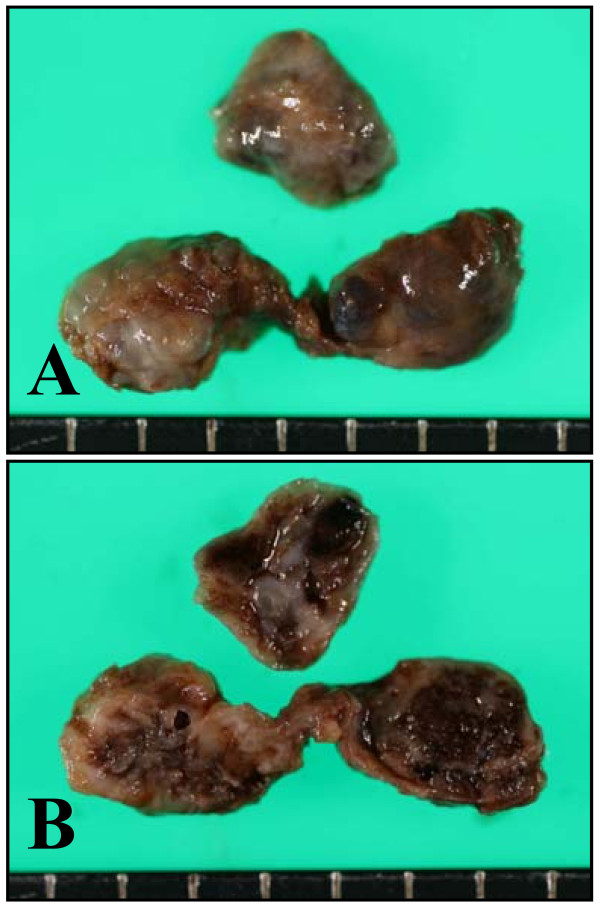
**Macroscopic findings of the lesion**. **(A) **The lesion had a serpentine appearance. **(B) **The cut surface of the lesion. It had a blackish area filled with blood and a whitish area.

A 43-year-old Japanese man presented with multiple chondromas and hemangiomas. His disease had been diagnosed as Maffucci syndrome. His available clinical information, however, was limited. One of the hemangiomas was removed and examined pathologically. Macroscopically, the lesion showed a serpentine appearance (Figure 1A). The cut surface of the lesion showed a blackish area filled with blood and a whitish area (Figure 1B).

**Figure 2:**
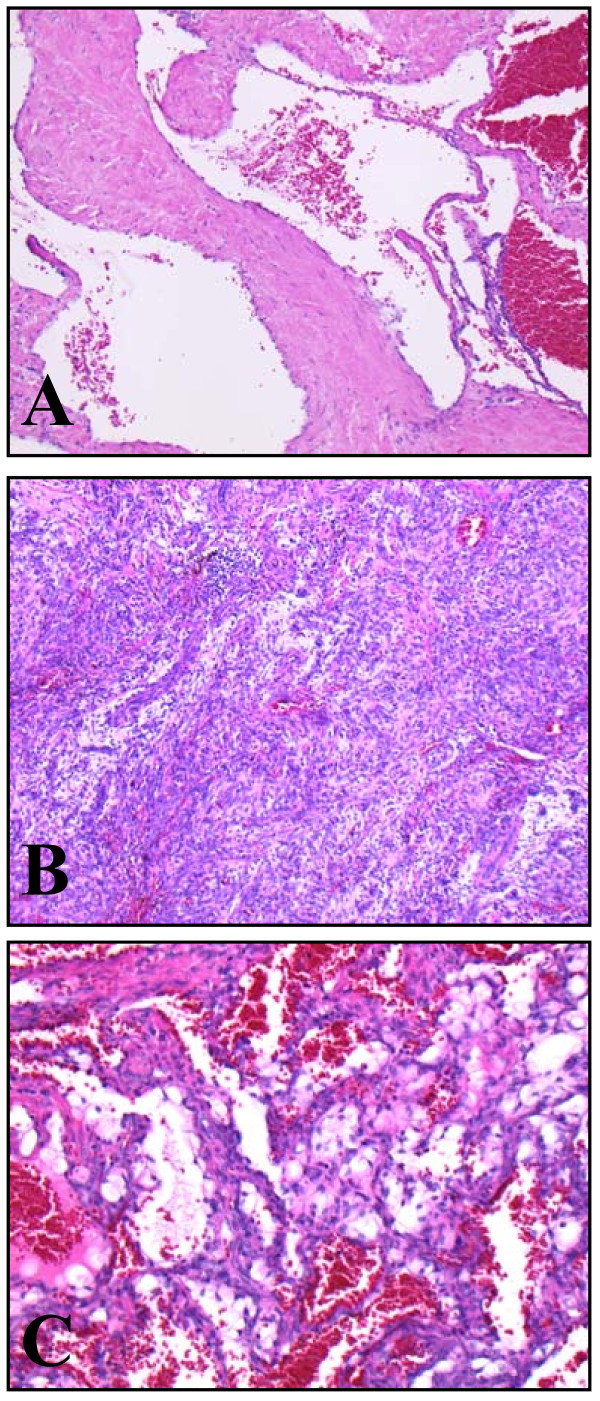
**Microscopic image showing the lesion**. **(A) **Cavernous hemangiomatous component (hematoxylin and eosin stain; original magnification, ×100). **(B) **Component of spindle cell hemangioma (hematoxylin and eosin stain; original magnification, ×100). **(C) **Sheets of vacuolated cells (hematoxylin and eosin stain; original magnification, ×200).

The morphological picture was an admixture of cavernous hemangioma (Figure 2A) and spindle cell hemangioma (Figure 2B) without cytological atypia or mitosis. Sheets of vacuolated endothelial cells were also observed (Figure 2C). In the cavernous component, organized thrombosis was observed (image not shown). No epithelioid hemangiomatous area was found.

## Discussion

Most patients with Maffucci syndrome present at birth or in early childhood with hemangiomas varying in size from a few millimeters to several centimeters which are typically located in the dermis or subcutaneously on the distal parts of the limbs. Hemangiomas, however, may also be found in internal organs [2]. The most common vascular lesions to occur in association with Maffucci syndrome are spindle cell hemangiomas, although occasional cases of lymphangiomas, arteriovenous malformations, and angiosarcomas have also been reported [3]. Thus, treatment for Maffucci syndrome should be aimed at early detection of malignant transformation as well as at symptom relief [4]. The problem could be how to do the follow-up of multiple hemangiomas located in the internal organs, how to analyze their histology, and which lesions to biopsy. In this patient, a histologically benign composite type hemangioma (cavernous and spindle cell hemangioma) was found, and no sarcomatous area was observed. Follow-up of the patient has revealed no signs of malignant transformation for years. Careful follow-up is, however, needed.

## Conclusion

In conclusion, a rare case of hemangioma associated with Maffucci syndrome has been reported, with a focus on the pathologic findings of the submitted tissue.

## Consent

Written informed consent was obtained from the patient for publication of this case report and any accompanying images. A copy of the written consent is available for review by the Editor-in-Chief of this journal.

## Competing interests

The author declares that they have no competing interests.

## Authors' contributions

TK performed the histological examination, analyzed the case, and wrote the manuscript.
